# Supplementary figures and images for: A Splice Intervention Therapy for Autosomal Recessive Juvenile Parkinson’s Disease Arising from Parkin Mutations
Source: Int J Mol Sci. 2020 Oct 1;21(19):7282. doi: 10.3390/ijms21197282 (PMC7582384; doi:10.3390/ijms21197282)

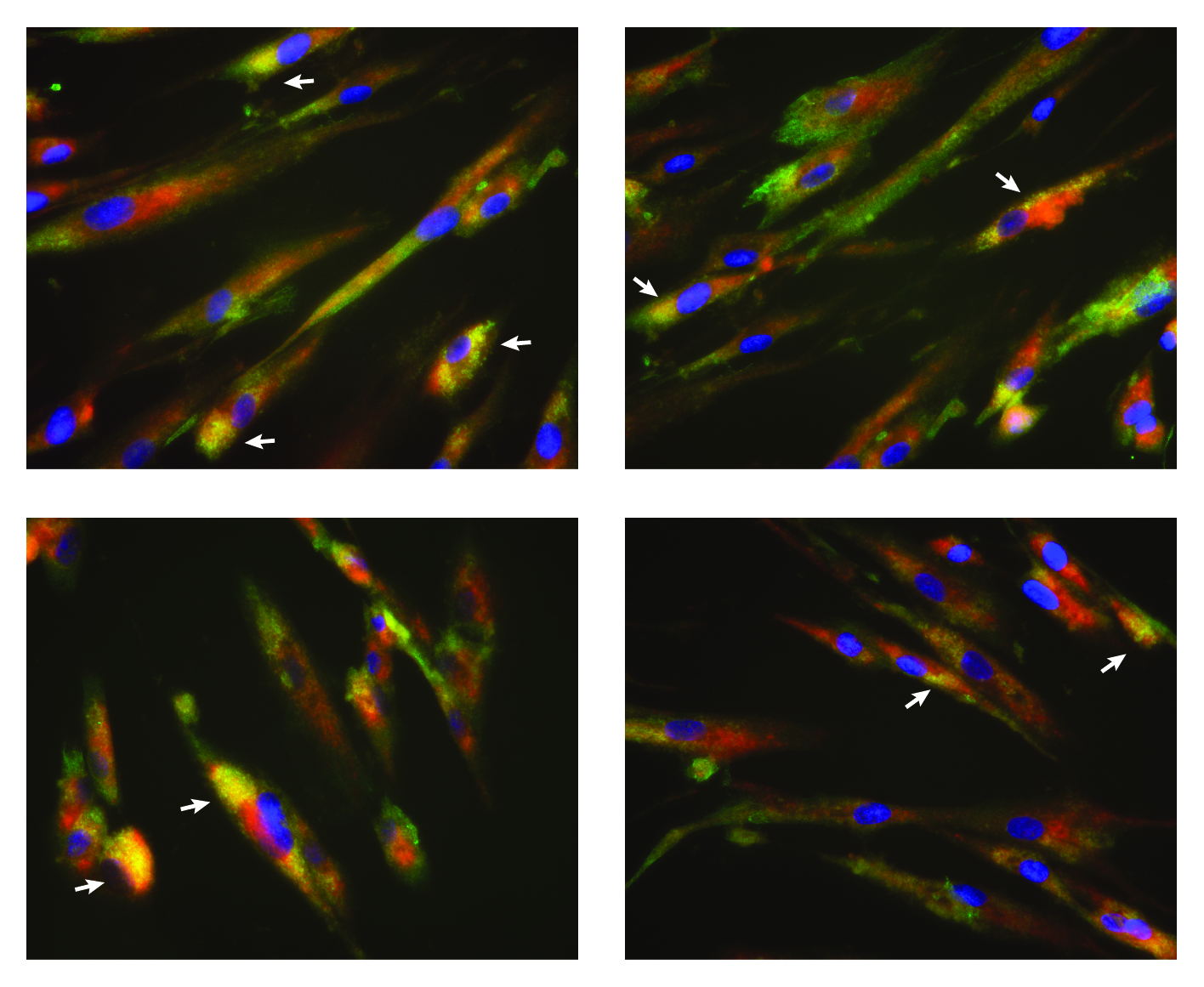

Supplement: Supplementary file 1 [file ijms-21-07282-s001.zip › Supplementary information/Supplementary Figure 3.jpg]

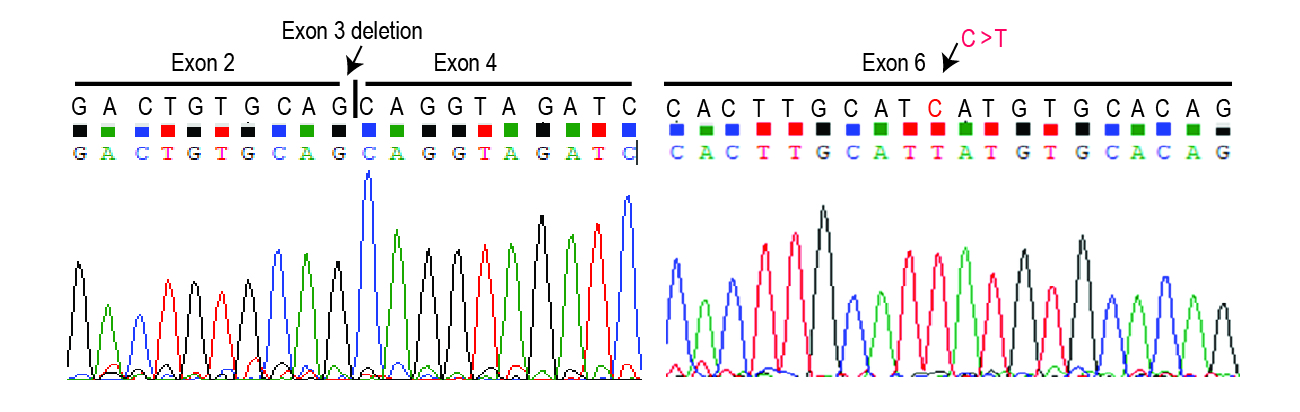

Supplement: Supplementary file 1 [file ijms-21-07282-s001.zip › Supplementary information/Supplementary Figure1.jpg]
