# Supplementary material for: A Splice Intervention Therapy for Autosomal Recessive Juvenile Parkinson’s Disease Arising from Parkin Mutations
Source: Int J Mol Sci. 2020 Oct 1;21(19):7282. doi: 10.3390/ijms21197282 (PMC7582384; doi:10.3390/ijms21197282)
Supplement: Supplementary file 1 [file ijms-21-07282-s001.zip › Supplementary information/Supplementary data.doc]

**Supplementary Figures**

Supplementary Figure 1. Sanger sequencing of RT-PCR products amplified from the patient fibroblasts. The *PRKN* mutations in a PD patient were confirmed by Sanger sequencing, with an exon 3 deletion detected on one allele and a single base substitution (c.719 C> T) in exon 6 on the second allele. Sanger sequencing was done using a forward primer in exon 3 that only showed the mutant base in exon 6, and a reverse primer in exon 8 that detected the exon 3 deletion.

Supplementary Figure 2. Over-exposed western blot image. Overexpoused (21 second exposure, 30 Sum in the accumulation mode) western blot showing no truncated parkin protein was detected in the untreated and gene-tool control AO treated samples.

Supplementary Figure 3. Immunofluorescent merge images showing colocalisation of truncated functional parkin protein and depolarized mitochondria. Arrows indicate the colocalisation of induced parkin protein and depolarized mitochondria.
